# Cutaneous and Venereal Memoranda

**Published:** 1878-01

**Authors:** 


*Cutaneous and Venereal Memoranda.* By HENRY G. PIFFARD, A. M., M. D., and GEORGE HENRY FOX, A. M., M. D. New York: Wm. Wood & Co. 1877. Buffalo: H. H. Otis. pp. 279.

This little volume consists of forty-five chapters, one-half being devoted to cutaneous diseases, the remainder to venereal diseases. The object of the work is well shown by this extract from the preface. "The fact that many students are unable during their pupilage to procure voluminous works on special subjects has induced the Authors to prepare the present volume. In doing so they have endeavored to inculcate principles rather than to elaborate details, and to present the facts in as compact a form as possible." The object they had in view has been attained. It will be of use to the student. C.

